# Dissociation of functional and structural plasticity of dendritic spines during NMDAR and mGluR-dependent long-term synaptic depression in wild-type and fragile X model mice

**DOI:** 10.1038/s41380-020-0821-6

**Published:** 2020-07-01

**Authors:** Aurore Thomazeau, Miquel Bosch, Sofia Essayan-Perez, Stephanie A. Barnes, Hector De Jesus-Cortes, Mark F. Bear

**Affiliations:** 1 The Picower Institute for Learning and Memory, Massachusetts Institute of Technology, Cambridge, MA 02139 USA; 2 Department of Basic Sciences, Universitat Internacional de Catalunya, 08195 Sant Cugat del Vallès, Spain; 3 Institute for Biomedical Research Agustí Pi i Sunyer, 08036 Barcelona, Spain

**Keywords:** Neuroscience, Physiology, Diseases

## Abstract

Many neurodevelopmental disorders are characterized by impaired functional synaptic plasticity and abnormal dendritic spine morphology, but little is known about how these are related. Previous work in the *Fmr1*^*-/y*^ mouse model of fragile X (FX) suggests that increased constitutive dendritic protein synthesis yields exaggerated mGluR5-dependent long-term synaptic depression (LTD) in area CA1 of the hippocampus, but an effect on spine structural plasticity remains to be determined. In the current study, we used simultaneous electrophysiology and time-lapse two photon imaging to examine how spines change their structure during LTD induced by activation of mGluRs or NMDA receptors (NMDARs), and how this plasticity is altered in *Fmr1*^*-/y*^ mice. We were surprised to find that mGluR activation causes LTD and AMPA receptor internalization, but no spine shrinkage in either wildtype or *Fmr1*^*-/y*^ mice. In contrast, NMDAR activation caused spine shrinkage as well as LTD in both genotypes. Spine shrinkage was initiated by non-ionotropic (metabotropic) signaling through NMDARs, and in wild-type mice this structural plasticity required activation of mTORC1 and new protein synthesis. In striking contrast, NMDA-induced spine plasticity in *Fmr1*^*-/y*^ mice was no longer dependent on acute activation of mTORC1 or *de novo* protein synthesis. These findings reveal that the structural consequences of mGluR and metabotropic NMDAR activation differ, and that a brake on spine structural plasticity, normally provided by mTORC1 regulation of protein synthesis, is absent in FX. Increased constitutive protein synthesis in FX appears to modify functional and structural plasticity induced through different glutamate receptors.

## Introduction

Structure and function are closely related at all levels of organization in the nervous system, from circuits to neurons to individual synapses. Excitatory synapses in the mammalian brain are predominantly located on dendritic spines, which act as biochemical compartments that allow the independent integration of presynaptic inputs. Synapses can undergo long-term changes in their transmission efficiency depending on the patterns of neuronal activity and, at the same time, activity can persistently modify spine morphology [1]. These forms of functional and structural plasticity are believed to represent the fundamental building blocks of learning and memory [2] and they are usually correlated: spines enlarge during long-term synaptic potentiation (LTP) [3] and shrink during long-term depression (LTD) [4–6]. The mechanisms underlying functional plasticity have been extensively studied, but much less is known about the mechanisms of structural plasticity and how both are interconnected.

Neurodevelopmental disorders like fragile X (FX) syndrome are characterized by deficits in functional plasticity and also by alterations in spine morphology [7]. However, little is known about how these changes might be related. In the hippocampus, LTD can be induced by activation of the NMDA-type glutamate receptor (NMDAR) or metabotropic glutamate receptor 5 (mGluR5). NMDAR-dependent LTD (NMDAR-LTD) can be induced by low-frequency synaptic stimulation (LFS) [8, 9] or by brief application of the selective agonist NMDA (N-methyl-D-aspartate) [10] and is associated with shrinkage of dendritic spines [5, 9, 11]. mGluR5-dependent LTD (mGluR-LTD) can be induced by patterned synaptic stimulation [12] or by application of the mGluR5 agonist DHPG ((*S*)-3,5-dihydroxyphenylglycine) [13, 14]. NMDAR-LTD and mGluR-LTD both occur in hippocampal area CA1 and are expressed by the internalization of AMPA receptors [15, 16], but they are mechanistically distinct and do not show mutual occlusion [14, 17]. Distinctive properties of mGluR-LTD include a dependence upon the rapid translation of dendritic mRNAs [12], but in the *Fmr1*^*-/y*^ mouse model of FX lacking the mRNA-binding protein FMRP, LTD is exaggerated [18] and no longer sensitive to protein synthesis inhibitors [19]. Interestingly, dendritic spines have also been reported to be altered in brain tissue from *Fmr1*^*-/y*^ rodents and FX patients [20]. These findings provoke the questions of how spines might change during mGluR-LTD [21, 22] and how structural plasticity differs in wild-type (WT) and *Fmr1*^*-/y*^ mice.

Therefore, our initial objectives in this study were to determine: (1) what kind of structural changes in dendritic spines are associated with mGluR-LTD in the hippocampus and (2) whether this structural plasticity is altered in the *Fmr1*^*-/y*^ mouse model of FX. We hypothesized that mGluR-LTD would show correlated spine shrinkage in WT mice, as seen in NMDAR-LTD [11], and that this shrinkage would be exaggerated in the *Fmr1*^*-/y*^ model, as seen in functional mGluR-LTD [18]. Our results, however, were inconsistent with these predictions. While NMDA indeed induced synaptic weakening that correlated with spine shrinkage, LTD induced by mGluR5 activation failed to correlate with a persistent change in spine structure in either WT or *Fmr1*^*-/y*^ mice.

The dissociation of functional and structural plasticity following mGluR5 activation inspired additional experiments to differentiate the signaling requirements for NMDAR-LTD and spine shrinkage. We found that application of compounds that block ion flux through the NMDAR completely blocked LTD, but had no effect on spine shrinkage. On the other hand, compounds that inhibit the mTORC1 signaling pathway or protein synthesis had no effect on LTD, but strongly inhibited spine shrinkage in WT mice. Interestingly, spine shrinkage in the *Fmr1*^*-/y*^ mouse no longer required mTORC1 or protein synthesis, suggesting increased abundance of a normally rate-limiting protein for structural plasticity. Consistent with this interpretation, increasing basal protein synthesis in WT slices by pre-incubation with an mGluR5 positive allosteric modulator (PAM) rendered spine shrinkage in response to NMDA insensitive to a protein synthesis inhibitor.

Thus, spines shrink in response to “metabotropic” NMDAR signaling rather than mGluR5, and this effect is indeed exaggerated in the *Fmr1*^*-/y*^ mouse compared with WT, but only under conditions when protein synthesis is inhibited. The data suggest that a brake on spine plasticity provided by regulation of mTORC1-dependent protein synthesis is missing in FX.

## Materials and methods

### Animals

*Fmr1*^*-/y*^ mice [23] and Thy1-GFP mice [24] were obtained from Jackson Laboratories, Maine, USA (stock # 003025 and # 011070, respectively). Both strains were backcrossed onto a C57BL/6J background for at least six generations at MIT and were subsequently maintained on a congenic C57BL/6J background by regular additional backcrossing. Experimental cohorts consisted of male littermates that were P25-P35 at the time of experiments. Cohorts were obtained from Thy1-GFP homozygous males × Thy1-GFP homozygous/*Fmr1* heterozygous female breeders. Mice were group housed with littermates and maintained on a 12:12 h light:dark cycle. All experiments were performed blind to genotype using age-matched littermate controls during the light phase. The Institutional Animal Care and Use Committee at Massachusetts Institute of Technology approved all experimental techniques.

### Hippocampal slices

Animals were deeply anesthetized through isoflurane inhalation (AErrane; Baxter Pharmaceuticals) and then decapitated. Acute dorsal hippocampal slices (350 μm thick) were prepared in ice-cold dissection buffer containing (in mM): NaCl 87, sucrose 75, KCl 2.5, NaH_2_PO_4_ 1.25, NaHCO_3_ 25, CaCl_2_ 0.5, MgCl_2_ 7, ascorbic acid 1.3, and D-glucose 10 (saturated with 95% O_2_/5% CO_2_). Immediately after slicing, the CA3 region was removed. Slices were recovered in artificial cerebrospinal fluid (ACSF) containing (in mM): NaCl 124, KCl 5, NaH_2_PO_4_ 1.23, NaHCO_3_ 26, CaCl_2_ 2, MgCl_2_ 1 and D-glucose 10 (saturated with 95% O_2_/5% CO_2_) at 32.5 °C for at least 3 h before recording.

### Hippocampal slice culture and gene transfection

Hippocampal organotypic slice cultures were prepared from postnatal day 6–7 rats as described [25]. Slices were cultured at 35 °C on interface membranes (Millipore) and fed with MEM media containing 20% horse serum and (in mM), D-glucose 27, NaHCO_3_ 6, CaCl_2_ 2, MgSO_4_ 2, HEPES 30, 0.01% ascorbic acid and 1 µg/ml insulin. pH was adjusted to 7.3 and osmolality to 300–320 mOsm. Slices were biolistically transfected (BioRad) after 5–7 days in vitro (DIV) with a plasmid expressing DsRed2 (Clontech) and a plasmid expressing SEP-GluA2 (kind gift of R. Malinow), both under CAG promoter.

### Electrophysiology

Field potential recordings were performed in a submersion chamber, perfused with ACSF (2–3 ml/min) at 30 °C. fEPSPs were recorded in CA1 stratum radiatum with extracellular electrodes filled with ACSF. Baseline responses were evoked by stimulation of the Schaffer collaterals at 0.033 Hz with a two-contact cluster electrode (FHC, Bowdoin, ME) using a 0.2 ms stimulus yielding 40–60% of the maximal response. Field recordings were filtered at 2 kHz, digitized at 50 kHz and analyzed using pClamp10 (Axon Instruments). The initial slope of the response was used to assess changes in synaptic strength. Data were binned per minute and normalized to the baseline and are presented as group mean ± S.E.M. Functional LTD was quantified by comparing the average response 50–60 min after NMDA, low-frequency stimulation (LFS; 1 Hz 15 min), or DHPG application to the average of the last 10 min of baseline. Experiments showing >5% of drift variation during baseline, calculated by fitting a linear regression line for the 30 min of baseline, were excluded from analysis. Paired-pulse facilitation was induced by applying two pulses at different inter-stimulus intervals. Facilitation was measured by the ratio of the fEPSP slope of response to stimulus 2 to stimulus 1.

### Two-photon laser-scanning microscopy

Time-lapse fluorescence imaging was carried out simultaneously with electrophysiological recordings using a two-photon microscope (Prairie Technologies Ultima system attached to an Olympus BX-51WI) equipped with a mode-lock femtosecond-pulse Ti:Sapphire laser (Chameleon, Coherent). Green and red fluorescent proteins were simultaneously excited at 930 nm. Images were taken with a 60 × 0.9 NA objective lens, and a digital zoom of ×5.65 every 4 min for the 30 min baseline and up to 1 h after LTD induction. For organotypic slices, imaging was performed at DIV 8–9 on primary or secondary dendrites from the proximal part of the main apical dendrite of CA1 pyramidal neurons. For both acute and organotypic slice experiments, well-isolated neurons with moderate GFP signal were carefully selected, with evident healthy dendritic morphology and no signs of fluorescent aggregates. At the end of each experiment, we reconfirmed that the neuron retained its healthy dendritic morphology. Data were normalized to the baseline and are presented as group mean ± S.E.M. Spine structural plasticity was quantified by comparing the average response 50–60 min after NMDA or DHPG application to the average of the last 10 min of baseline.

### LTD induction and pharmacological reagents

NMDAR-dependent LTD was induced by applying NMDA (20 µM) for 3 min, and mGluR-LTD was induced by applying R,S-DHPG (50 µM) for 5 min. In pharmacological pretreatment experiments, slices were pre-incubated with the respective drug for 40 min before the beginning of baseline recordings, and then kept in bath throughout the entire experiment (except Supplementary Fig. S5 and Fig. 5d). NMDA, MK-801 (40 µM) were purchased from Sigma. R,S-DHPG, D-AP5 (50 µM), 7-CK (100 µM), U0126 (20 µM) were purchased from Tocris Biosciences. Rapamycin (20 nM) was purchased from LC labs. Fresh bottles of DHPG were prepared as a 100× stock in H_2_O, divided into aliquots and stored at −20 °C. Fresh stocks were made once a week. CHX (60 µM) and CDPPB (3-Cyano-*N*-(1,3-diphenyl-1*H*-pyrazol-5-yl)benzamide; 10 µM) were purchased from Tocris Biosciences. CDPPB was made up weekly in DMSO; CHX was fresh each experimental day.

### Image analysis

For every time point, a series of 512 × 512 pixel XY-scanned images (Z-Series) was taken every 1 µm of tissue depth, for 20 µm of depth in total. The maximal fluorescence intensity of the Z-Series was summed to obtain a single collapsed image (Z-stack) for every time point. During each experiment, 24 Z-stacks were collected 4 min apart, over 30 min of baseline and 1 h of LTD induction. These 24 Z-stacks were compiled into a movie montage, to track fluorescence intensity in the *X*, *Y*, and *Z* dimensions in each frame. The montage was aligned using the StackReg function in Fiji/ImageJ (by Rasband, W.S., U. S. National Institutes of Health) [26]. On average, 15 spines on multiple dendritic regions were followed throughout the montage. The only criteria for selection of spines were that their entire morphology was clearly visible over a dark background throughout all frames in the movie; they were required to be located on healthy dendritic regions that were visually isolated, and to have clearly resolved heads and necks, irrespective of their size and shape. This way, selected spines included diverse sizes and diverse morphologies, such as thin, thick, or mushroom shapes, but excluding dim filopodia and stubby spines. Spines were excluded if they overlapped with neighboring spines or other dendrites in any frame of the movie. A constant 20 × 20 pixel circular region of interest (ROI) was outlined around the spine, including the spine head and half of the spine neck. Within this ROI, the total integrated fluorescence intensity of the green and the red channels was calculated using ImageJ. Intensity values were background-subtracted and corrected for overall fluorescence fluctuations. The intensity of the dark background around the cell and spines was manually tracked at three locations in every frame to ensure the background ROI did not overlap with any dendrites or spines. For fluorescence fluctuation calculations, the intensities of five locations along a dendrite were measured and manually tracked across all frames. The background-corrected intensities of RFP and GFP signals were taken to be proportional to spine volume and the amount of fusion protein [27]. We confirmed that these values yielded similar results to those obtained from values of spine head area (data not shown). We only included experiments showing <7% of drift variation during baseline, calculated by fitting a linear regression line for the 30 min of baseline.

### Biochemistry

To mimic electrophysiology and imaging experiments, hippocampal slices were prepared as described above and transferred to sterile incubation chambers (440 µm polyester mesh, 15 mm insert, Costar 3478). Slices were recovered for 40 min at 32.5 °C followed by room temperature (RT) for 3–5 h in ACSF (saturated with 95% O_2_/5% CO_2_). MK-801 (40 µM) was then applied and kept in solution for the remainder of the experiment. After 30 min of MK-801 incubation, slices were transferred to 32.5 °C and after 15 min, baseline slices were flash frozen in liquid nitrogen. After another 15 min, other slices were exposed to NMDA or vehicle for 3 min, and 3 time points after NMDA were flash frozen: 0, 5, and 15 min (see Supplementary Fig. S6). All samples were stored at −80 °C until the day of immunoblotting. For immunoblotting, chambers containing each slice were submerged in ice-cold homogenization buffer A (20 mM Tris base, pH 7.4, 150 mM NaCl, 1 mM EDTA, 1 mM EGTA) with phosphatases and proteases cocktail inhibitors (EMD Millipore set I, 524624, set II, 524625 and set III, 539134) until thawed. Then, slices were individually transferred to 1.5 ml microcentrifuge tubes containing 50 µl of buffer A and stored on ice. Slices were then grinded using a pellet pestle (Kimble Kontes) for 10 s. This homogenate was centrifuged at 16,100 × *g* for 15 min and supernatant was then transferred to a clean tube for immunoblotting. Laemmli buffer (BioRad) with 2-mercaptoethanol (50 µl total) was added to each tube and incubated for 5 min at 100 °C to prepare samples for western blotting. Samples were stored at −80 °C until electrophoresis day. Protein extracts (30 µl) were loaded into 4–20% mini-PROTEAN TGX gels (BioRad) and ran for 55 min at 150 V. We then transferred the protein to a 0.2 µm nitrocellulose membrane using the Trans-Blot Turbo transfer system (BioRad) manufacturer protocol for mixed molecular weight proteins. Membranes were blocked using the TBS Odyssey Blocking buffer (Li-cor) for 1 h at RT followed by incubation of target protein antibody (diluted in blocking buffer with 0.2% tween 20) overnight at 4 °C. The next day, membranes were washed 5 min 3× at RT using TBS (BioRad) with 0.1% Tween 20 (TBST). Membranes were then incubated in secondary antibody corresponding to the primary antibody species (1:5000 IRDye 800 CM Donkey anti-Rabbit, 926-32213 or 1:15,000 IRDye 680RD Donkey anti-Mouse, 926–68072 from Licor) for 1 h at RT. This was followed by three washes of 5 min in TBST, and then by another three washes of 5 min in TBS. Images were collected using ChemiDoc MP auto-exposure user protocol for each fluorophore (BioRad). Phosphorylated proteins were blotted first followed by stripping off the membranes using NewBlot Nitro Stripping Buffer (Licor) and re-blotted for total proteins. For densitometric analysis (quantification of protein bands), we used ImageLab version 6.0 (BioRad). Antibodies and concentrations used: 1:1000 phospho-mTOR (Ser2448, CST 2971), 1:1000 mTOR (CST 2972), 1:1000 phospho-S6 Ribosomal protein (Ser 235/236, CST 4856), 1:1000 S6 Ribosomal protein (CST 2217), 1:1000 phospho-ERK1/2 (Thr202/Tyr204, CST 9101), 1:1000 ERK1/2 (CST 9102).

### Statistical analyses

All values are expressed as mean ± S.E.M. Statistical significance was set at the 95% confidence level (two-tailed) and calculated using Prism 7.0 (GraphPad software). Multiple comparisons (genotype and drug effect) were analyzed using two-way ANOVA with post-hoc Bonferroni’s test. Single comparisons were made with paired Student’s *t* test to calculate differences between the average of the 50–60 min interval post NMDA/DHPG treatment and the baseline, and unpaired Student’s *t* test to calculate differences between the average of the 50–60 min time interval. Pharmacological or genetic experiments were statistically compared with their corresponding vehicle or WT controls. Experiments were performed blind to the genotype and in an interleaved manner, each with a different slice but within the same experimental day. The order of control and experimental conditions were randomized. Statistics were performed using N as animal, with each animal represented by one slice. Variance between genotypes or drug/vehicle treatment groups were similar for each type of experiment. For each figure, average time course of mean fEPSPs, mean spine volume, or SEP fluorescence was normalized to baseline.

## Results

### Dissociation of functional and structural plasticity during mGluR-dependent LTD

**Fig. 1:**
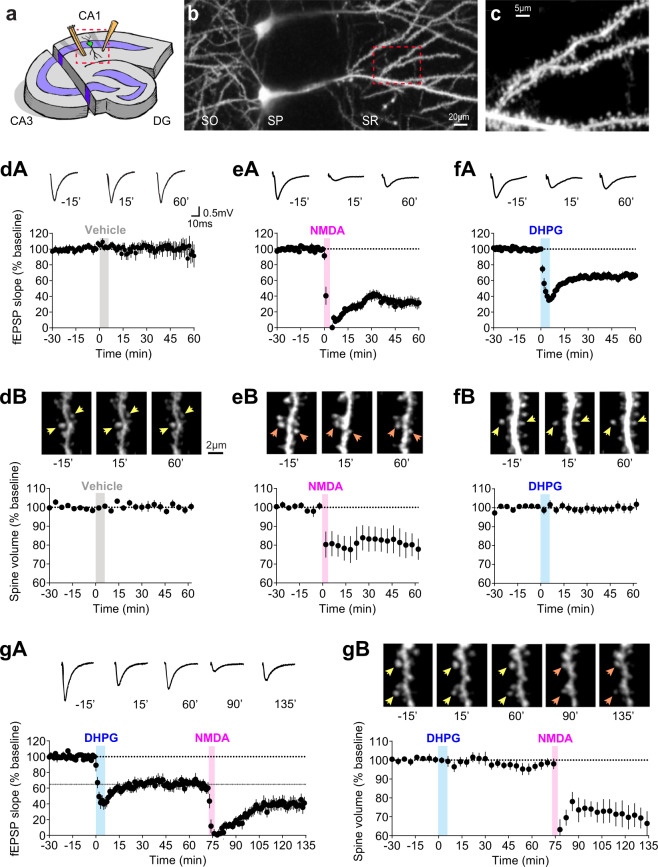
Dissociation between functional and structural plasticity during mGluR-LTD. **a** Extracellular field recordings and time-lapse two-photon imaging were simultaneously performed in the CA1 region of acute Thy1-GFP mouse hippocampal slices with CA3 removed to ensure that agonists acted specifically on receptors on CA1 neurons. **b** Two-photon image of CA1 pyramidal neurons. SO: Stratum Oriens; SP: Stratum Pyramidale; SR: Stratum Radiatum. **c** Magnified image of the red-squared region in **b** showing SR proximal dendrites with dendritic spines. **d**–**g** Time-course of averaged fEPSP slope responses (**A**) and averaged dendritic spine volume (**B**) normalized to baseline (dashed lines). Representative fEPSP traces and images of dendritic spines are shown at 3 time points: 15 min before, 15 and 60 min after LTD induction. Sequential experiments (**gA, B**) include two more time points: 90 min and 135 min after induction. Scale bars apply to all panels. Yellow arrows indicate unaltered spines, orange arrows indicate shrinking spines. **dA** Bath application of vehicle (aCSF, 5 min, gray bars) to hippocampal slices did not alter fEPSP slope (10 min period immediately before vehicle: 102.38 ± 1.81% of total baseline; 50–60 min period after vehicle: 99.40 ± 7.41% of total baseline, *n* = 10 animals; n.s. *p* = 0.6635, paired *t*-test). **dB** Vehicle did not induce any persistent structural change in spine volume (before: 99.50 ± 0.48%; after vehicle: 99.70 ± 1.71%, *n* = 10; n.s. *p* = 0.9077, paired *t*-test). **eA** Bath application of NMDA (20 µM, 3 min, magenta bar) induced LTD of fEPSPs (10 min period immediately before NMDA: 99.88 ± 1.65% of total baseline; 50–60 min period after NMDA: 31.78 ± 5.53% of total baseline, *n* = 9 animals; ****p < 0.0001, paired *t*-test). **eB** NMDA induced a long-term decrease in the volume of spines (before: 99.51 ± 0.68%; after NMDA: 83.72 ± 5.29%, *n* = 9; *p = 0.0235, paired *t*-test). **fA** A Bath application of the mGluR agonist DHPG (50 µM, 5 min, blue bar) induced LTD of fEPSPs (before: 99.39 ± 0.76%; after DHPG: 65.3 0 ± 3.35%, *n* = 16; ***p < 0.0001, paired *t*-test). **fB** DHPG did not induce any persistent change in spine volume (before: 100.40 ± 0.52%; after DHPG: 100.30 ± 2.46%, *n* = 14; n.s. *p* = 0.9687, paired *t*-test). Sequential induction of mGluR-LTD and NMDAR-LTD. **gA** Application of DHPG followed by NMDA induced additional LTD (*n* = 8; before: 99.85 ± 1.28%; after DHPG: 65.62 ± 5.28%, ***p = 0.005, paired t-test; before NMDA: 65.62 ± 5.28% (lower dashed line); after NMDA: 39.96 ± 7.37% of initial baseline; **p = 0.0022 with respect to before-NMDA, paired *t*-test). **gB** DHPG application did not change spine volume (before: 101.10 ± 1.10%; after DHPG: 97.78 ± 1.90%; n.s. *p* = 0.128, paired *t*-test) but subsequent NMDA application elicited a robust long-term spine shrinkage in the same population of spines (before: 97.78 ± 1.90%; after NMDA: 68.94 ± 6.57%; ***p = 0.00066, paired *t*-test). This subsequent spine shrinkage (volume decreased by 32.47 ± 6.0%) tended to be more pronounced than the spine shrinkage caused by the single NMDA application (**eB**), but this difference did not achieve statistical significance.

We wanted to simultaneously study structural and functional plasticity associated with the induction of NMDAR-dependent and mGluR-dependent LTD in hippocampal neurons. For this purpose, we prepared acute hippocampal slices from P25–35 Thy1-GFP mice. These mice express GFP in a random subset of neurons, which allows the clear visualization of dendritic spines on well-isolated dendrites [24]. We used two-photon time-lapse fluorescence microscopy to image the apical proximal dendrites in the stratum radiatum region of CA1 pyramidal neurons (Fig. 1a–c). At the same time, we placed stimulating and recording electrodes in the region flanking the imaged dendrite (Fig. 1a). We stimulated the Schaffer collateral axons every 30 s and recorded extracellular field excitatory postsynaptic potentials (fEPSPs). To induce widespread LTD of excitatory synapses we used well established chemical induction protocols. NMDAR-LTD was induced by NMDA (20 μM, 3 min) [10] and mGluR-LTD by bath application of DHPG (50 μM, 5 min) [12]. With area CA3 cut away (Fig. 1a), direct stimulation of CA1 with a highly selective agonist enables reproducible induction of each distinct form of LTD across a large population of synapses. In control experiments with vehicle treatment, both fEPSPs (Fig. 1dA) and spine volume (Fig. 1dB) were stably maintained over time. However, as previously reported, application of NMDA induced a strong long-lasting depression of fEPSPs (Fig. 1eA) [10], which was accompanied by a correlated long-lasting reduction of dendritic spine volume (Fig. 1eB) [11]. Similarly, DHPG treatment induced a robust and stable depression of fEPSPs [14] (Fig. 1fA), but surprisingly, we did not observe a net reduction of spine volume up to 1 h following drug application (Fig. 1fB). Additional analysis of the persistent change in size as a function of the initial spine size revealed no systematic differences between populations that could account for our findings: large and small spines both showed structural shrinkage after NMDA but not after DHPG or vehicle (Supplementary Fig. S1). Although spine volume changed after NMDA, neither treatment significantly modified spine density (data not shown).

Although a failure to observe spine shrinkage during mGluR1-dependent LTD was reported in the cerebellum [21], this finding was unexpected in the hippocampus where LTD is dependent on mGluR5 [22]. We therefore wanted to confirm that in the same preparation in which DHPG was without effect, NMDA could still induce spine changes. Thus, we designed an experiment in which mGluR-LTD was induced first using DHPG, and then NMDAR-LTD was induced with NMDA, as we monitored the same population of dendritic spines. This experiment is feasible because mGluR- and NMDAR-LTD do not mutually occlude [14, 17]. As expected, we were able to sequentially induce mGluR-LTD and NMDAR-LTD in the same population of stimulated synapses, and in agreement with our initial findings, spine volume decreased after NMDA; DHPG had a negligible effect (Figs. 1g, S2). Spine shrinkage induced by NMDA following DHPG exposure (Fig. 1Gb) appeared to be exaggerated compared with NMDA alone (Fig. 1eB), but this difference did not achieve statistical significance.

A trivial reason for why mGluR-LTD induction might not be accompanied by persistent spine shrinkage is that it is expressed presynaptically. Although a mechanistically distinct presynaptic form of mGluR-LTD has been reported in slices from early postnatal rodents [28], there is a general consensus that both NMDAR-LTD and mGluR-LTD are expressed postsynaptically in P25–35 mice. Nevertheless, to ensure there was no major presynaptic component in our LTD induction protocols, we monitored paired pulse facilitation (PPF). LTD that arises from a reduction in glutamate release probability is accompanied by increased PPF [29]. No significant difference in PPF was detected after the application of NMDA or DHPG at any of the inter-stimulus intervals tested (Supplementary Fig. S3a, b) and there was no correlation between LTD magnitude and a change in PPF (Supplementary Fig. S3c, d). We therefore conclude that both LTD forms are expressed primarily via postsynaptic mechanisms in our preparations.

### DHPG induces the internalization of AMPA receptors without shrinking dendritic spines

Chemical induction protocols were used to maximize the number of synapses affected, and a clear effect on spine volume was observed after NMDA that correlates with LTD. However, the absence of lasting structural plasticity following DHPG conceivably could be due to a failure to image the appropriate population of spines. As both NMDAR- and mGluR-LTD are expressed postsynaptically by the internalization of AMPARs [16, 30], we turned to organotypic slice cultures and used fluorescently-tagged AMPAR trafficking as a way to optically measure the degree of functional LTD at each individual spine. We transfected cultured hippocampal slices with the GluA2 subunit of the AMPAR fused to Synapto-Ecliptic-pHluorine (SEP). This fusion protein is fluorescent when receptors are expressed on the plasma membrane, but the fluorescence is quenched when receptors are internalized into acidic vesicles [31, 32]. By co-transfecting neurons with the red fluorescent protein DsRed2 together with SEP-GluA2 we could simultaneously measure changes in spine volume and surface expression of AMPARs in individual spines.

**Fig. 2:**
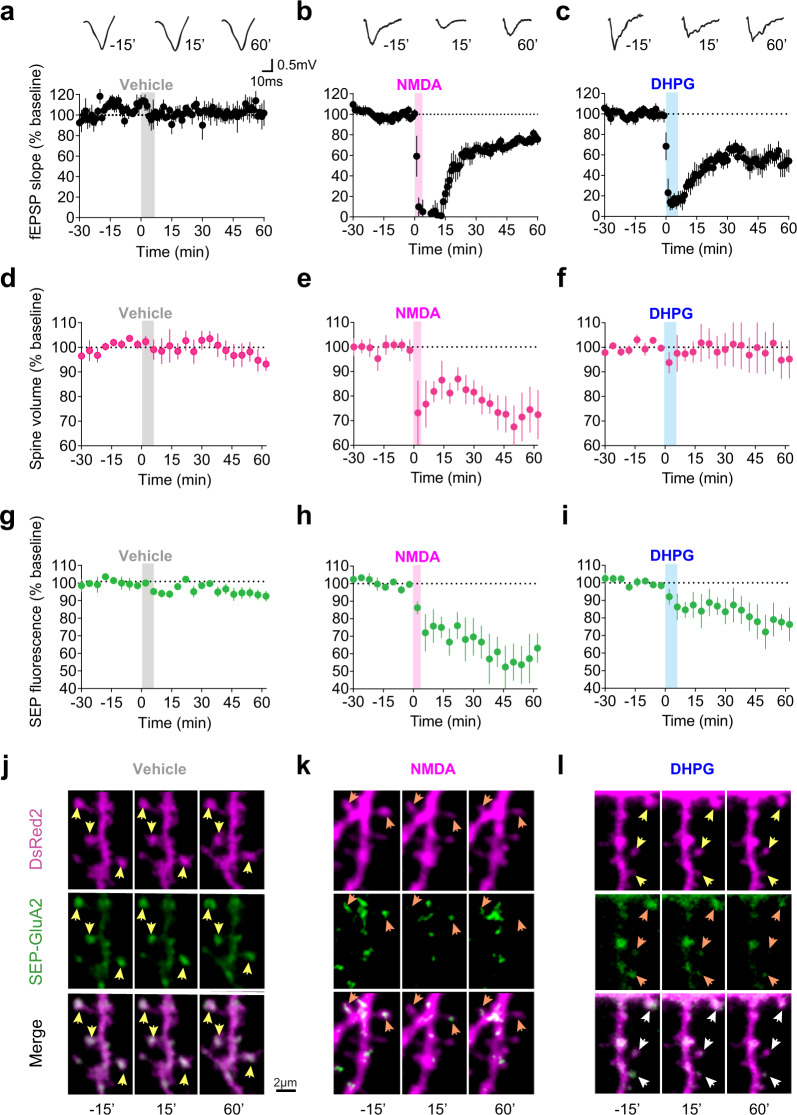
AMPA receptor internalization is associated with both mGluR- and NMDAR-LTD. The content of AMPAR in the membrane surface of dendritic spines was monitored by measuring the fluorescence of SEP fused to the GluA2 subunit. Simultaneous field recordings and two-photon imaging were performed in the CA1 region of organotypic cultured hippocampal slices (CA3 removed) co-transfected with DsRed2 and SEP-GluA2. **a**–**c** Time-course of averaged fEPSP responses normalized to baseline. Representative fEPSP traces are shown at three time points: 15 min before, 15 and 60 min after LTD induction. Scale bar applies to all panels. **a** Application of vehicle (aCSF, 5 min, gray bars) did not alter fEPSP slope (10 min before vehicle: 104.28 ± 3.06% of total baseline; 50–60 min after vehicle: 105.03 ± 6.28%, *n* = 4, n.s. *p* = 0.916, paired *t*-test). **b** Bath application of NMDA (20 µM, 3 min) induced LTD of fEPSPs (10 min before NMDA: 99.25 ± 1.75% of total baseline; 50–60 min after NMDA: 75.62 ± 3.25%, *n* = 6, **p = 0.0023, paired *t*-test). **c** Bath application of DHPG (50 µ, 5 min) induced LTD of fEPSPs (before: 100.5 ± 3.38%; after DHPG: 56.38 ± 9.05%, *n* = 6; **p = 0.0048, paired *t*-test). **d**–**f** Time-course of the averaged spine volume (measured from DsRed2 fluorescence intensity) normalized to baseline. **d** Vehicle did not induce any persistent structural change in spine volume (before: 101.98 ± 0.25%; after vehicle: 95.72 ± 2.79%, *n* = 4; n.s. *p* = 0.096, paired *t*-test). **e** Spine volume persistently decreased upon NMDA application (before: 100.01 ± 1.09%; after NMDA: 71.47 ± 4.14%, *n* = 6; ***p = 0.0007, paired *t*-test). **f** DHPG did not induce any persistent structural change in spine volume (before: 100.6 ± 0.65%; after DHPG: 97.27 ± 8.35%, *n* = 6; n.s. *p* = 0.7221, paired *t*-test). **g**–**i** Time-course of the averaged fluorescence intensity of SEP-GluA2 in the spine, normalized to baseline. SEP fluorescence does not change after (**g**) vehicle application (before: 99.26 ± 2.78%; after vehicle 93.69 ± 2.86%, *n* = 4; n.s. *p* = 0.090, paired *t*-test), but persistently decreased after (**h**) NMDA application (before: 98.98 ± 0.77%; after NMDA: 57.37 ± 9.66%, *n* = 6; **p = 0.0075, paired *t*-test) and after (**i**) DHPG application (before: 99.64 ± 0.90%; after DHPG: 76.29 ± 7.61%, *n* = 6; *p = 0.0234, paired *t*-test). **j**–**k** Representative two-photon images of segments of secondary apical dendrites showing DsRed2 (magenta), SEP-GluA2 (green) and merged (yellow) fluorescence, at three times points (15 min before, 15 after, and 60 min after vehicle application or LTD induction). Scale bar applies to all panels. Yellow arrows indicate spines with no change in volume or AMPAR, orange arrows indicate spines showing AMPAR internalization or shrinkage, white arrows indicate spines showing AMPAR internalization but no shrinkage.

We induced both forms of LTD in these organotypic slices and obtained results comparable to those observed in acute slices. In control experiments with vehicle treatment, both fEPSPs (Fig. 2a) and spine volume (Fig. 2d) were stably maintained over time. However, NMDA induced LTD of extracellular field potentials (Fig. 2b) and a significant decrease in spine volume (Fig. 2e). Induction of mGluR-LTD (Fig. 2c), on the other hand, had no net effect on spine volume (Fig. 2f). Thus, the dissociation of functional and structural plasticity following activation of mGluR5 was confirmed using two different experimental approaches: acute adolescent mouse hippocampal slices (Fig. 1fB) and cultured rat hippocampal slices (Fig. 2f). We also tried inducing structural plasticity with DHPG in a number of additional preparations of rat and mouse hippocampus, and mouse visual cortex, and consistently failed to observe lasting spine shrinkage (Supplementary Fig. S4).

Despite the absence of structural change, we did observe a significant decrease of SEP fluorescence in dendritic spines after DHPG application (Fig. 2i, l), indicating that AMPARs were efficiently removed from the spine surface following mGluR-LTD induction. Application of NMDA was also accompanied by a decrease of SEP fluorescence in dendritic spines (Fig. 2h, k), likely reflecting both AMPAR internalization and depolarization-induced acidification of the cytoplasm [33]. In control experiments, we confirmed that application of vehicle did not result in any appreciable change in SEP fluorescence (Fig. 2g, j). These results strongly suggest that mGluR-LTD is expressed postsynaptically by the internalization of AMPARs in spines that fail to undergo long-term structural plasticity. Taken together, these findings indicate that functional postsynaptic plasticity and spine structural changes are dissociable—one does not follow automatically from the other.

### A metabotropic effect of NMDAR activation elicits structural but not functional plasticity

Lack of correlation between functional and structural changes after mGluR5 activation suggests that these two forms of plasticity are regulated by different intracellular mechanisms. Similarly, although functional and structural changes are usually correlated after NMDAR activation, their intracellular mechanisms have also been shown to diverge [34]. Recent studies have suggested that in addition to their ionotropic actions, NMDARs exert metabotropic effects that contribute to LTD [35, 36]; however, this conclusion is controversial [37]. As mGluR-LTD is induced by a pure metabotropic mechanism, we investigated the ionotropic and non-ionotropic (metabotropic) requirements for functional and structural plasticity following NMDAR activation.

**Fig. 3:**
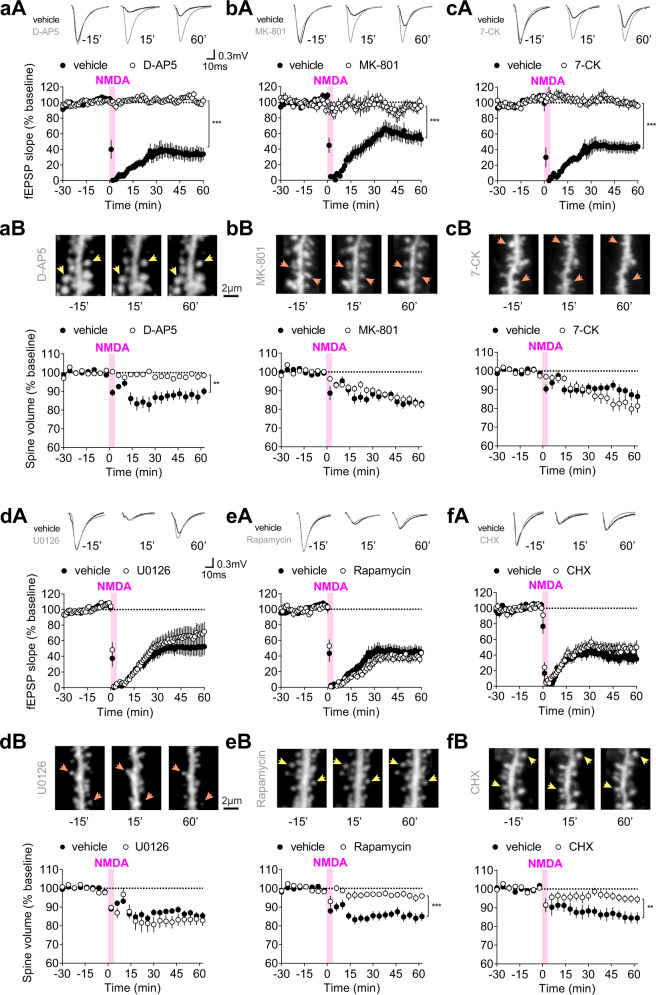
Metabotropic action of NMDAR, mTOR pathway and protein synthesis are required for structural plasticity but not functional NMDAR-LTD in WT mice. The mechanisms underlying structural and functional plasticity were studied using simultaneous field recordings and two-photon imaging of dendritic spines, performed in stratum radiatum of CA1 in acute slices of WT (Thy1-GFP) mice during continuous bath application of various pharmacological treatments. Representative fEPSP traces (vehicle in black, drug in gray) and spine images during drug application are shown at three time points: 15 min before, 15 and 60 min after LTD induction. Scale bars apply to all panels. Yellow arrows indicate unaltered spines, orange arrows indicate shrinking spines. **a**–**c** Pharmacological study of metabotropic and ionotropic NMDAR actions. **aA** The competitive NMDAR antagonist D-AP5 (50 µM) blocked functional NMDA-induced LTD (vehicle, black circles: 37.12 ± 8.44% of baseline, *n* = 7; D-AP5, white circles: 103.18 ± 2.82%, *n* = 8; ****p < 0.0001 with respect to vehicle, unpaired *t*-test). **aB** D-AP5 treatment prevented the structural change in dendritic spine volume following NMDA application (vehicle, black circles: 87.53 ± 3.16%, *n* = 7; D-AP5, white circles: 98.32 ± 1.58%, *n* = 8; **p = 0.0072 with respect to vehicle, unpaired *t*-test). **bA** The NMDAR open-channel blocker MK-801 (40 µM) prevented induction of functional LTD (vehicle, black circles: 55.51 ± 8.10%, *n* = 9; MK-801, white circles: 94.83 ± 5.08%, *n* = 9; ***p = 0.0008 with respect to vehicle, unpaired *t*-test). **bB** Unlike functional NMDAR-LTD, MK-801 treatment had no effect on spine structural plasticity (vehicle, black circles: 83.40 ± 1.64%, *n* = 9; MK-801, white circles: 84.20 ± 2.02%, *n* = 9; n.s. *p* = 0.7629 with respect to vehicle, unpaired *t*-test). **cA** 7-CK (100 µM) competitively antagonized the NMDAR co-agonist (glycine) site and blocked expression of functional LTD (vehicle, black circles: 44.56 ± 6.30%, *n* = 7; 7-CK, white circles: 97.53 ± 4.14%, *n* = 8; ****p < 0.0001 with respect to vehicle, unpaired *t*-test). **cB**, In contrast, 7-CK treatment had no effect on spine shrinkage (vehicle, black circles: 87.29 ± 2.43%, *n* = 7; 7-CK, white circles: 81.90 ± 2.91%, *n* = 8; n.s. *p* = 0.1867 with respect to vehicle, unpaired t-test). **d**–**f** Pharmacological study of intracellular signaling pathways. **dA** The MEK inhibitor U0126 (20 µM) did not affect functional NMDAR-LTD (vehicle, black circles: 51.62 ± 11.08% of baseline, *n* = 12; U0126, white circles: 67.24 ± 12.58%, *n* = 11; n.s. *p* = 0.3602, unpaired *t*-test), and (**dB**) did not alter NMDA-induced dendritic spine shrinkage (vehicle, black circles: 85.92 ± 1.92%, *n* = 12; U0126, white circles: 83.43 ± 3.02%, *n* = 11; n.s. *p* = 0.4863, unpaired *t*-test). **eA** The mTORC1 inhibitor rapamycin (20 nM) did not affect functional NMDAR-LTD (vehicle, black circles: 44.87 ± 7.75%, *n* = 10; Rapamycin, white circles: 38.70 ± 5.34%, *n* = 12; n.s. *p* = 0.5087, unpaired *t*-test), but (**eB**) significantly reduced the NMDA-induced decrease of spine volume (vehicle, black circles: 84.89 ± 2.45%, *n* = 10; Rapamycin, white circles: 96.01 ± 1.44%, *n* = 12, ***p = 0.00059, unpaired *t*-test). **fA** The protein synthesis inhibitor cycloheximide (CHX, 60 µM) did not alter functional NMDAR-LTD (vehicle, black circles: 37.32 ± 5.50%, *n* = 14; CHX, white circles: 48.43 ± 6.00%, *n* = 20; n.s. *p* = 0.2021, unpaired *t*-test), but (**fB**) CHX treatment significantly reduced the NMDA-induced spine shrinkage (vehicle, black circles: 85.38 ± 2.88%, *n* = 14; CHX, white circles: 95.12 ± 2.02%, *n* = 20; **p = 0.0073, unpaired *t*-test).

We first verified that the competitive NMDAR antagonist D-AP5 was able to completely prevent both functional LTD and structural plasticity induced by NMDA application (Fig. 3aA, B). This finding confirms that ligand binding to the glutamate binding sites on the NMDAR is required for both types of plasticity [10, 11]. To test whether blocking the ion flux through NMDARs was sufficient to prevent functional or structural NMDAR-LTD, we treated hippocampal slices for 60 min with the open-channel blocker MK-801. This compound blocks ion flux without affecting glutamate or NMDA binding. We found that MK-801 pretreatment completely prevented induction and expression of functional NMDAR-LTD (Fig. 3bA). Inhibition of LTD was also observed in experiments in which MK-801 was present only during baseline stimulation, which would produce activity-dependent block of synaptic NMDARs only, and in experiments in which synaptic LFS was used to induce LTD instead of NMDA application (Supplementary Fig. S5). However, MK-801 had no effect on induction of spine structural plasticity (Fig. 3bB). We confirmed this result with 7-CK, a competitive antagonist for the glycine site on NMDARs, which also blocks ion flux without affecting glutamate or NMDA binding. Like MK-801, 7-CK completely prevented functional LTD (Fig. 3cA) without affecting structural plasticity (Fig. 3cB). These findings indicate that spine shrinkage relies on a metabotropic effect of NMDAR activation, whereas functional LTD depends on ion flux through the receptor. The fact that morphology changes in the absence of ion flux rules out spurious causes of spine shrinkage that might accompany massive depolarization caused by NMDA.

### Structural plasticity requires mTORC1 pathway activation and de novo protein synthesis

Activation of NMDARs stimulates several signaling kinases, such as Ras and Rap, upstream of ERK (extracellular signal-regulated kinase). Since ERK1/2 has been implicated in fragile X [38] and plays a critical role in synaptic plasticity [39] including mGluR-LTD [40], we examined the involvement of this signal transduction pathway in functional and structural plasticity during NMDAR-LTD. We applied the MEK1/2-ERK1/2 inhibitor U0126 1 h before NMDA treatment and found that neither functional NMDAR-LTD (Fig. 3dA) nor structural plasticity (Fig. 3dB) were affected. NMDARs also activate alternative intracellular mechanisms including the mTOR signaling cascade [41–43]. We therefore tested the involvement of the mTOR pathway in NMDAR-LTD by applying the mTORC1 inhibitor rapamycin. Rapamycin had no effect on functional NMDAR-LTD (Fig. 3eA), but significantly reduced the magnitude of spine shrinkage produced after NMDA application (Fig. 3eB). Thus, functional and structural plasticity following NMDA are doubly dissociable: MK-801 and 7-CK selectively block one form (LTD), and rapamycin selectively blocks the other (spine shrinkage).

The mTOR pathway is classically associated with the control of protein synthesis [44]. Dendritic protein synthesis is required for mGluR-LTD, the late phases of NMDAR-LTD and LTP, and regulation of homeostatic plasticity [12, 45, 46]. Moreover, local protein synthesis is also needed for the late phase of structural change of dendritic spines after LTP induction [27, 47]. Therefore, we next tested the requirement of de novo protein synthesis by applying the translation inhibitor cycloheximide (CHX) 80 min before NMDA treatment. CHX had no effect on synaptic transmission or the induction or expression of LTD (Fig. 3fA), but it significantly reduced spine plasticity caused by the NMDA application (Fig. 3fB). These data suggest that structural plasticity of dendritic spines during NMDAR-LTD is dependent on the ongoing synthesis of new proteins under the control of the mTORC1 pathway.

These findings motivated us to ask if stimulation of NMDARs in the absence of ion flux could elicit a detectable increase in mTORC1 activity during spine plasticity. NMDA was applied in the presence of MK-801 and slices were flash frozen at various time points for biochemical analysis of changes in phosphorylation of either mTOR or the downstream reporter ribosomal protein S6. There was no indication of acute activation of this pathway, or ERK1/2, by NMDA during the early, rapamycin-sensitive phase of spine shrinkage, although we did see mTOR phosphorylation increased 15 min after NMDA treatment (Supplementary Fig. S6). Although we cannot rule out the possibility that this biochemical assay is insufficiently sensitive to detect rapid increases in synaptic mTORC1-dependent protein synthesis, which does not always correlate with mTOR or S6 phosphorylation, the data are also compatible with the alternative hypothesis that constitutive rather than acutely stimulated protein synthesis is necessary for rapid spine plasticity.

### Signaling requirements for structural plasticity differ in *Fmr1*^*-/y*^ mice

**Fig. 4:**
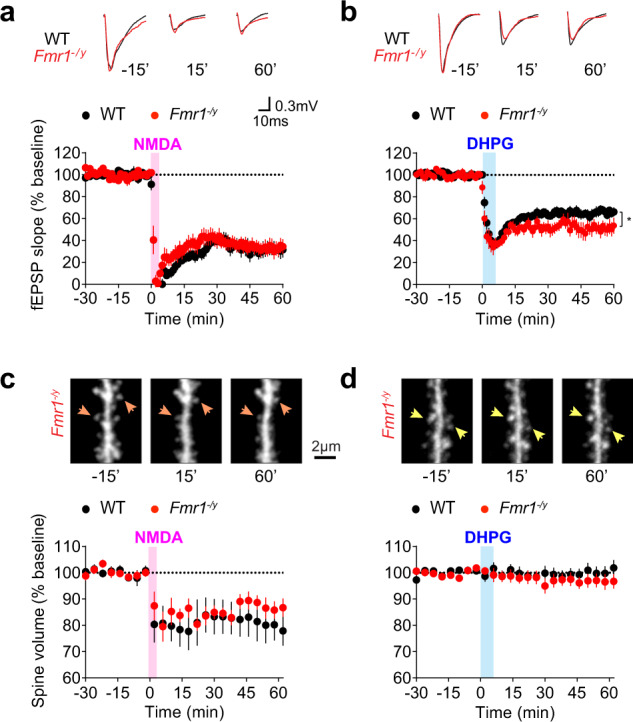
Spine structural plasticity is not altered in fragile X mice. Extracellular field recordings and time-lapse two-photon imaging of dendritic spines were performed simultaneously in stratum radiatum of CA1 in acute slices of *Fmr1*^*-/y*^ mice interleaved with slices of their WT littermates (both Thy1-GFP). Comparison of the functional and structural plasticity induced by bath application of NMDA or DHPG in both genotypes. Representative fEPSP traces (WT in black, *Fmr1*^*-/y*^ in red) and images of dendritic spines in *Fmr1*^*-/y*^ mice are shown at 3 time points: 15 min before, 15 and 60 min after LTD induction. Scale bars apply to all panels. Yellow arrows indicate unaltered spines, orange arrows indicate shrinking spines. **a** NMDA-induced LTD in hippocampus from *Fmr1*^*-/y*^ mice was comparable in magnitude to that observed in WT littermates (WT, black circles: 31.78 ± 5.53%, *n* = 9; *Fmr1*^*-/y*^, red circles: 32.97 ± 6.22%, *n* = 8; n.s. *p* = 0.8877 with respect to WT, unpaired *t*-test). **b** DHPG-induced LTD in *Fmr1*^*-/y*^ mice was significantly greater than in WT mice (WT, black circles: 65.29 ± 3.35%, *n* = 16; *Fmr1*^*-/y*^, red circles: 51.25 ± 5.44%, *n* = 11; *p = 0.0285, unpaired *t*-test). c NMDA induced a significant shrinkage of dendritic spines from *Fmr1*^*-/y*^ mice similar to WT mice (WT, black circles: 83.72 ± 5.29%, *n* = 9; *Fmr1*^*-/y*^, red circles: 88.71 ± 3.31%, *n* = 8; n.s. *p* = 0.4492, unpaired *t*-test). **d** DHPG did not induce any persistent structural changes in spines from *Fmr1*^*-/y*^ or from WT mice (WT, black circles: 100.32 ± 2.46%, *n* = 14; *Fmr1*^*-/y*^ red circles: 93.364 ± 2.48%, *n* = 13; n.s. *p* = 0.3078, unpaired *t*-test).

We next examined functional and structural plasticity in the *Fmr1*^*-/y*^ model of fragile X. Under baseline slice imaging conditions, we observed no difference in *Fmr1*^*-/y*^ spine density compared with WT (5.14 ± 0.23 spines/10 µm in *Fmr1*^*-/y*^ vs 5.46 ± 0.22 in WT, *p* = 0.312, unpaired *t*-test) and a slight but significant increase in spine length (2.42 ± 0.06 µm in *Fmr1*^*-/y*^ vs 2.26 ± 0.05 in WT, *p* = 0.0482, unpaired *t*-test), consistent with published findings [7, 48]. However, because mGluR-LTD and protein synthesis are exaggerated in *Fmr1*^*-/y*^ mice, we hypothesized that structural plasticity would appear following DHPG and possibly be exaggerated following NMDA. As expected from previous studies, NMDAR-LTD was no different in the *Fmr1*^*-/y*^ mice and mGluR-LTD was significantly increased [18] (Fig. 4a, b). However, contrary to our hypothesis, structural plasticity in the *Fmr1*^*-/y*^ mice resembled WT. Spine shrinkage occurred following NMDA application but not DHPG, and there was no difference between genotypes (Fig. 4c, d). As in WT, there was no significant change in spine density after either treatment (data not shown).

**Fig. 5:**
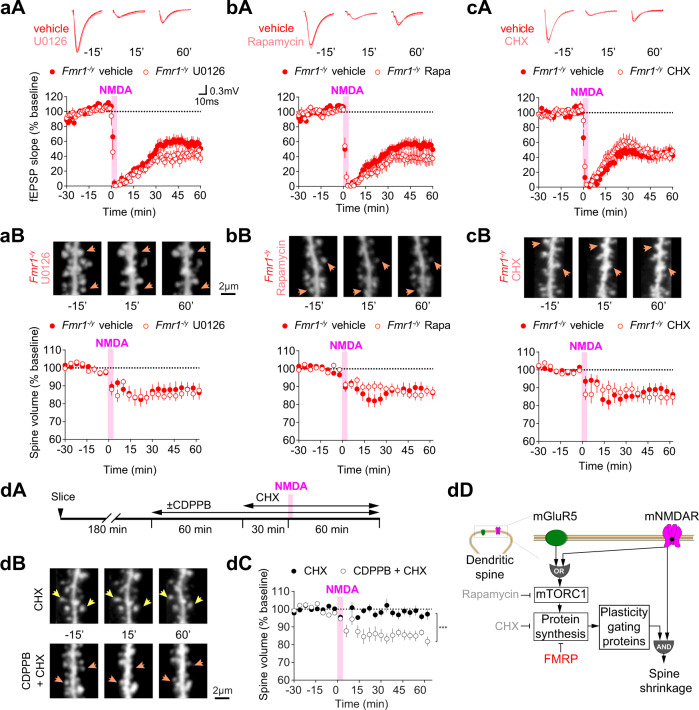
A fragile X structural plasticity phenotype is revealed in the presence of rapamycin or cycloheximide. The mechanisms underlying structural and functional plasticity in fragile X were studied, similarly to Fig. 3, using extracellular field recordings and time-lapse two-photon imaging of dendritic spines in stratum radiatum of CA1 in acute slices of *Fmr1*^*-/y*^ mice interleaved with slices of their WT littermates (both Thy1-GFP). **a**–**c** Representative fEPSP traces (*Fmr1*^*-/y*^ in red, *Fmr1*^*-/y*^ with drug in pink) and images of spines in *Fmr1*^*-/y*^ mice during the application of intracellular signaling inhibitor are shown at three time points: 15 min before, 15 and 60 min after NMDAR-LTD induction. Scale bars apply to all panels. Yellow arrows indicate unaltered spines, orange arrows indicate shrinking spines. **aA** U0126 (20 µM) did not affect functional NMDAR-LTD in slices from *Fmr1*^*-/y*^ mice (*Fmr1*^*-/y*^ + vehicle, red circles: 56.22 ± 6.95%, *n* = 9; *Fmr1*^*-/y*^ + U0126, white circles: 42.69 ± 6.94%, *n* = 9; n.s. *p* = 0.1875, unpaired *t*-test), nor did it have any effect on NMDA-induced spine shrinkage in *Fmr1*^*-/y*^ mice (**aB** *Fmr1*^*-/y*^ + vehicle, red circles: 88.01 ± 3.48%, *n* = 9; *Fmr1*^*-/y*^ + U0126, white circles: 86.60 ± 3.02%, *n* = 9; n.s. *p* = 0.7641, unpaired *t*-test). **bA** Rapamycin (20 nM) did not affect functional NMDAR-LTD in Fmr1-/y mice (*Fmr1*^*-/y*^ + vehicle, red circles: 54.56 ± 8.01%, *n* = 10; *Fmr1*^*-/y*^ + rapamycin, white circles: 38.02 ± 8.67%, *n* = 12; n.s. *p* = 0.1828, unpaired *t*-test). **bB** Unlike WT littermates, rapamycin treatment had no effect on dendritic spine shrinkage induced by NMDA in Fmr1-/y mice (*Fmr1*^*-/y*^ + vehicle, red circles: 88.19 ± 3.12%, *n* = 10; *Fmr1*^*-/y*^ + Rapamycin, white circles: 86.92 ± 2.65%, *n* = 12; n.s. *p* = 0.7578, unpaired *t*-test. Two-way ANOVA, genotype versus treatment, provided significant interaction: *F* = 6.406, *p = 0.0154). **cA** CHX (60 µM) did not alter functional NMDAR-LTD in *Fmr1*^*-/y*^ mice (*Fmr1*^*-/y*^ + vehicle, red circles: 44.41 ± 6.79%, *n* = 15; *Fmr1*^*-/y*^ + CHX, white circles: 44.41 ± 5.41%, *n* = 15; n.s. *p* = 0.9997, unpaired *t*-test). **cB** Unlike WT littermates, CHX treatment had no effect on NMDA-induced dendritic spine shrinkage in *Fmr1*^*-/y*^ mice (*Fmr1*^*-/y*^ + vehicle, red circles: 88.25 ± 3.38%, *n* = 15; *Fmr1*^*-/y*^ + CHX, white circles: 84.98 ± 4.01%, *n* = 15; n.s. *p* = 0.5374, unpaired *t*-test. Two-way ANOVA, genotype versus treatment, provided significant interaction: *F* = 4.52, *p = 0.0376). **dA** Experimental timeline for the application of the mGluR5 positive allosteric modulator CDPPB and the protein synthesis inhibitor CHX before induction of NMDAR-LTD in WT slices. **dB** Representative images of dendritic spines after application of CHX with or without preincubation with CDPPB are shown at three time points: 15 min before, 15 and 60 min after NMDAR-LTD induction. Yellow arrows indicate unaltered spines, orange arrows indicate shrinking spines. **dC** Following preincubation with 10 μM CDPPB, the structural changes induced by NMDA are no longer blocked by 60 μM CHX (vehicle + CHX, black circles: 97.80 ± 2.14%, *n* = 9; CDPPB + CHX, white circles: 84.65 ± 2.51%, *n* = 8; ***p = 0.0006 with respect to vehicle + CHX, unpaired *t*-test). **dD** Schematic model on how mGluR5 and NMDAR regulate protein synthesis to gate structural plasticity at CA1 dendritic spines. According to this model, spine shrinkage requires metabotropic signaling by NMDARs (mNMDAR) *and* the presence of “plasticity gating proteins” that are rate-limiting. In WT under normal circumstances, the gate is opened by new protein synthesis in response to mNMDAR activation. In *Fmr1*^*-/y*^ mice, this gate is constitutively open due to translational de-repression. WT can be made to resemble *Fmr1*^*-/y*^ mice when protein synthesis is stimulated by prior continuous activation of mGluR5 with CDPPB. Activation of mGluR5 alone does not induce spine shrinkage because it requires the simultaneous activation of mNMDAR.

A peculiar finding in *Fmr1*^*-/y*^ mice is that mGluR-LTD, in addition to being exaggerated, is no longer blocked by CHX or other protein synthesis inhibitors [19, 49]. A similar phenotype has been observed in other mouse models of neurodevelopmental disorders associated with intellectual disability [50–52]. These findings are usually interpreted to mean that a protein species, normally limiting for plasticity in WT, is overabundant in the mutant mice due to increased basal protein synthesis. We therefore reexamined the signaling requirements for functional and structural plasticity following NMDA in the *Fmr1*^*-/y*^ mice. Similar to our findings in WT (Fig. 3dA, eA, fA), we observed no effect of ERK1/2, mTORC1, or protein synthesis inhibitors on functional NMDAR-LTD in the *Fmr1*^*-/y*^ mice (Fig. 5aA, bA, cA). Interestingly however, spine structural plasticity that is normally mTOR-dependent and protein synthesis-dependent in the WT (see Fig. 3eB, fB) is no longer sensitive to rapamycin or CHX in the *Fmr1*^*-/y*^ mice (Fig. 5aB, bB, cB). Thus, a structural plasticity phenotype downstream of metabotropic NMDAR activation is uncovered in the presence of inhibitors of mTORC1-dependent protein synthesis. This phenotype does not appear to be directly related to a difference in basal or stimulated mTOR activity in the slices (Supplementary Fig. S6). Rather, the data suggest that, in FX, the consequences of mTOR pathway activity are exaggerated due to de-repression of protein synthesis.

These findings indicate that spine structural plasticity should be added to the list of synaptic modifications that require new protein synthesis in WT, but not in *Fmr1*^*-/y*^ mice [19, 53]. Our data in WT mice suggest that the rate-limiting proteins that gate spine shrinkage at the time of induction are synthesized downstream of an mTORC1 signaling pathway. We therefore wondered if a pretreatment designed to increase the abundance of these hypothetical “plasticity gating proteins” might render WT synaptic structural plasticity insensitive to CHX at the time of induction, similar to what is observed at the *Fmr1*^*-/y*^ synapses. To examine this possibility, we pretreated WT slices for 60 min with the mGluR5-selective agonist and positive allosteric modulator CDPPB that has been shown to stimulate protein synthesis in slices [54] (Fig. 5dA). In WT CA1, mGluR5 signals via Homer to activate the phosphoinositide 3-kinase (PI3K)-Akt-mTOR pathway and initiate translation [55]. This experiment revealed that NMDA-induced spine shrinkage indeed is no longer CHX sensitive in WT slices pretreated with CDPPB (Fig. 5dB, C). These findings are consistent with the model that mGluR5 and metabotropic NMDARs converge on the mTORC1 signaling pathway to regulate abundance of plasticity gating proteins that are crucial for spine structural plasticity (Fig. 5dD). Translational de-repression in *Fmr1*^*-/y*^ mice impairs this regulatory mechanism.

## Discussion

It is well established that excitatory synapses in the hippocampus of juvenile mice and rats support two major forms of LTD, one triggered by the activation of NMDARs and the other by activation of mGluR5 [56]. One distinctive property of mGluR-LTD in WT mice and rats is a requirement for the rapid translation of mRNAs localized to CA1 dendrites [12]. However, in *Fmr1*^*-/y*^ mice that lack the translational repressor FMRP, mGluR-LTD is both increased and no longer protein synthesis-dependent [18, 19]. These findings suggested that mGluR5-stimulated protein synthesis is exaggerated under basal conditions and the checkpoint proteins that are normally rate-limiting for LTD are overexpressed in fragile X. In contrast, NMDAR-LTD, for which there is no requirement in WT for rapid protein synthesis, is unaffected in the *Fmr1*^*-/y*^ mice.

With these data as a foundation, in the current study we set the goal to test a prediction that structural plasticity of dendritic spines downstream of mGluR5 is also altered in fragile X and might contribute to reported differences in spine morphology [57]. However, our findings were surprising on multiple counts. First, we found that although LTD was reliably elicited with DHPG in WT (Fig. 1) and exaggerated in *Fmr1*^*-/y*^ mice (Fig. 4), it failed to correlate with a change in dendritic spine structure, even in experiments in which we confirmed the loss of postsynaptic AMPARs (Fig. 2). These findings argue that any spine morphological defects in hippocampus of *Fmr1*^*-/y*^ mice are unlikely to be a direct consequence of altered mGluR-LTD, as had been conjectured [57]. Second, we discovered that although NMDA acts at the glutamate-binding sites on NMDARs (those blocked by D-AP5) to trigger both LTD and robust spine shrinkage, only LTD is blocked by inhibiting current flow through the channel (with MK-801 or 7-CK) (Fig. 3a–c). Third, we found that the metabotropic effect of NMDA that manifests as spine shrinkage in the absence of ion flux is blocked in WT mice by inhibitors of both mTORC1 and protein synthesis (Fig. 3d–f). Fourth, we discovered that while NMDAR-LTD and spine shrinkage appear identical in WT and *Fmr1*^*-/y*^ mice under control conditions, there is no longer a requirement for mTORC1 or protein synthesis for spine plasticity in the absence of FMRP (Figs. 4,  5). The data are consistent with the idea that checkpoint proteins are overexpressed in fragile X, but now in the novel context of structural plasticity downstream of metabotropic NMDAR signaling. Consistent with this hypothesis, boosting basal protein synthesis in WT by pre-treatment with CDPPB phenocopies FX. Investigation of metabotropic NMDAR regulation of protein synthesis and structural plasticity therefore offers the potential to identify novel therapeutic targets for treatment of fragile X.

### No net shrinkage of dendritic spines during mGluR-LTD

We studied acutely prepared hippocampal slices at an age (P25-35) when mGluR-LTD is robust, postsynaptically expressed, dependent upon new protein synthesis in WT, and exaggerated in *Fmr1*^*-/y*^ mice. To induce LTD, we briefly applied the mGluR5 agonist DHPG [13, 14]. One advantage of this “chem-LTD” approach, besides high reproducibility and receptor specificity, is that a large population of synapses is affected simultaneously. However, despite confirming in every experiment that LTD was indeed induced in stratum radiatum, we failed to observe a lasting net change in the volume of dendritic spines on CA1 pyramidal cell apical dendrites. Similar observations were made in cultured hippocampal slices in which we confirmed AMPAR internalization after DHPG treatment in individual dendritic spines. We also failed to observe structural plasticity after DHPG in acutely prepared *Fmr1*^*-/y*^ slices, as well as cultured slices from rat hippocampus or mouse visual cortex. Our failure to observe spine shrinkage after DHPG is not explained by an inability to detect spine plasticity, as evidenced by the robust effects of NMDA in the same preparations. We therefore conclude that under multiple experimental conditions, spine shrinkage does not accompany LTD triggered by activation of mGluR5 in the hippocampus. Interestingly, a similar dissociation of postsynaptic functional and structural plasticity has also been observed in the cerebellum, where a mechanistically distinct form of LTD [58] is triggered by activation of mGluR1 [21].

Our findings appear to conflict with those of a study by Ramiro-Cortes, et al. [22] in which spine shrinkage was observed after DHPG in cultured mouse hippocampal slices. However, a key methodological difference is that in their experiments DHPG was applied to slices that included area CA3 as well as CA1. Thus, in addition to direct actions of the agonist on mGluRs in CA1, there was the possibility that myriad other mechanisms could be recruited via electrical activity originating in CA3. Indeed, the spine shrinkage that was observed in CA1 following DHPG was prevented by application of tetrodotoxin, demonstrating a requirement for sodium-dependent action potentials. The most parsimonious explanation for the different findings, therefore, is that in the study by Ramiro-Cortes et al. DHPG elicited CA3 spiking activity and the synaptic release of other factors that drove spine shrinkage. Interestingly, spine plasticity in that study was also prevented by inhibitors of protein synthesis (cycloheximide and anisomycin) reminiscent of what we observe after NMDA. However, unlike our observations, the spine plasticity observed by Ramiro-Cortes was not affected by AP5. These findings may be reconciled if spiking triggered the release of ligands for receptors other than mGluR5 and NMDAR on dendritic spines to drive structural plasticity.

The possible involvement of mGluRs in spine plasticity was also suggested in another study by Oh, et al. [9] using cultured slices of rat hippocampus. They found that low-frequency uncaging of glutamate at identified spines would induce both LTD and a reduction of spine volume, similar to what we observe after NMDA in our preparation. Furthermore, both the functional and structural plasticity was blocked by a competitive antagonist of the glutamate binding site on NMDARs, consistent with our findings. However, they found that shrinkage of large (but not small) spines was additionally inhibited by negative allosteric modulators (NAMs) of mGluR5 and mGluR1. In contrast, we were unable to detect a selective vulnerability of large spines to structural modification following DHPG (Supplementary Fig. S1). Our data do not exclude the possibility that mGluR5 or mGluR1 contribute to plasticity of large spines following activation of NMDARs, but they do indicate that activation of mGluR5 and mGluR1 alone (with DHPG) is not sufficient to drive net spine shrinkage under conditions where LTD is readily demonstrated. Since Oh et al. found that glutamate uncaging fails to trigger shrinkage of either large or small spines in the presence of a selective NMDAR antagonist, their findings and ours are in good agreement. Our data are consistent with the possibility that prior exposure to DHPG, which can stimulate new protein synthesis, may “prime” some spines for shrinkage after NMDA (cf. Fig. 1eB, gB). This effect is most clearly observed when structural plasticity is studied in the presence of CHX (Fig. 5d).

Interestingly, Wiergert and Oertner [59] also failed to observe rapid spine shrinkage following optogenetic low-frequency stimulation of Schaffer collateral synapses in CA1, but did document gradual spine elimination 1–7 days later. We cannot rule out the possibility that spine shrinkage or elimination would be observed following DHPG over a longer time course. However, we can conclude that functional mGluR-LTD precedes by at least 1 h any structural changes, and therefore that functional and structural plasticity can be dissociated in hippocampal neurons.

### LTD induced by NMDA is triggered by ion flux but spine shrinkage depends on metabotropic NMDAR signaling

Data accumulated over many years have supported a model in which homosynaptic LTD is triggered by the modest or prolonged postsynaptic influx of Ca^2+^ through NMDARs [56]. However, this conclusion was challenged by recent studies in which LTD could still be induced in CA1 by low-frequency synaptic stimulation in the presence of the open-channel blocker MK-801 or the glycine site inhibitor 7-CK, but was reliably blocked by the competitive glutamate-site antagonist AP5 [35, 36]. These findings suggested a functional consequence of metabotropic NMDAR activation by ligand binding that is independent of ion flux, for which there was some precedent [60, 61]. Subsequent studies confirmed that the NMDAR undergoes conformational changes in response to glutamate binding that can alter the interactions of enzymes tethered to the cytoplasmic domain of the receptor [62, 63]. However, the conclusion that signaling in the absence of current flow is sufficient to trigger functional LTD is controversial. Specifically, several laboratories have observed that NMDAR-LTD is indeed reliably blocked by MK-801 [37, 64, 65]. In agreement with these latter studies we found that MK-801 reliably blocked LTD induced by NMDA (Fig. 3b) or synaptic LFS (Supplementary Fig. S5b). We obtained similar results using the glycine-site inhibitor 7-CK which also blocks current flux without interfering with glutamate binding to the NMDAR. In addition, chem-LTD was blocked in experiments in which MK-801 was present only during baseline stimulation, which would produce activity-dependent block of synaptic NMDARs only (Supplementary Fig. S5a). Our observations do not support the hypothesis that metabotropic signaling is responsible solely for functional NMDAR-LTD.

Although ion flux also can play a role in spine shrinkage in some experimental preparations [66], we found that in the same slices where MK-801 reliably blocked LTD, it failed to prevent spine shrinkage after NMDA. Thus, our data support the conclusion that the structural plasticity that accompanies LTD is a consequence of non-ionotropic signaling [36]. Metabotropic NMDAR signaling is still a young field, and there is a great deal left to learn [67]. Several signaling mechanisms have already been implicated in previous investigations [36, 61, 63, 68], but we chose to focus here on pathways implicated in fragile X. We considered the hypotheses that either mTORC1 [69] or ERK1/2 [38, 40] signaling was involved in spine structural plasticity. Although the ERK1/2 pathway inhibitor U0126 had no effect on functional or structural plasticity, the mTORC1 inhibitor rapamycin strongly reduced spine shrinkage after NMDA without affecting functional LTD.

Since mTORC1 is a well-established regulator of new protein synthesis [44] we examined the effect of cycloheximide and again observed reduced spine plasticity with no effect on LTD (Fig. 3f). Previous work has established that *de novo* protein synthesis is required for the consolidation of the late phases of NMDAR-dependent LTP [70] and LTD [71] as well as for the late consolidation of structural changes after LTP induction [27, 47]. Our findings show it is also required for the rapid structural changes that accompany NMDAR-mediated LTD. Interestingly, a recent study in cultured hippocampal neurons observed that mTORC1 and protein synthesis are additionally involved in the early phases of spine enlargement following chemical induction (with glycine) of NMDAR-dependent LTP [72]. Clearly more work will be required to understand how NMDAR activation and protein synthesis specifically regulate spine morphology. One conclusion that fits the available data is that mTORC1 and protein synthesis play a permissive “gating” function, rather than an instructive role in the structural changes that follow NMDAR activation. This conclusion is consistent with our biochemical finding that in the absence of ion flux, NMDA does not appear to stimulate mTORC1 at the early time points when spine shrinkage is sensitive to rapamycin and cycloheximide. However, it is also possible that NMDAR-induced spine shrinkage in WT mice is mediated by mTORC1-eIF4E-dependent translation that can occur independently of mTOR or S6 phosphorylation [73, 74]. Settling this question will require a high-resolution analysis of protein synthesis at individual dendritic spines.

### A fragile X spine plasticity phenotype is revealed when mTORC1 or protein synthesis are inhibited

In WT mice, mGluR-LTD is considered to be a sensitive functional measure of local protein synthesis. Thus, the finding that mGluR-LTD is exaggerated in *Fmr1*^*-/y*^ mice fit nicely with the view that FMRP acts as a repressor of mRNA translation: increased mGluR5-dependent protein synthesis begets increased LTD [18]. Nevertheless, although this fragile X phenotype is reproducible, it can be subtle under control conditions (e.g., Fig. 4). However, the difference between WT and *Fmr1*^*-/y*^ is greatly accentuated when protein synthesis is blocked acutely with various translation inhibitors [19, 69]. A similar phenotype was subsequently shown in other mouse models of genetically defined intellectual disability when mGluR-LTD was studied in the presence of protein synthesis inhibitors [50–52]. The first working hypothesis proposed to account for these interesting findings was that there exist “LTD proteins”, normally under tight translational regulation via mGluR5 signaling, that are constitutively overexpressed in fragile X and related disorders. This concept was revised when it was discovered that the phenomenon of LTP priming, also linked to rapid mGluR5-dependent protein synthesis in WT, was likewise rendered insensitive to protein synthesis inhibitors in *Fmr1*^*-/y*^ mice [53]. Although the polarity of the modification was different, the implication again was that some rapidly turned over “plasticity-gating proteins” are necessary for various types of activity-dependent functional plasticity in WT animals, and these are overabundant in *Fmr1*^*-/y*^ mice.

The current findings now suggest a further modification of the concept to include NMDAR-dependent structural plasticity (Supplementary Fig. S7). We found that whereas spine plasticity after NMDA is substantially inhibited by rapamycin and cycloheximide in WT (Fig. 3), these treatments have no effect in *Fmr1*^*-/y*^ mice (Fig. 5). These findings underscore the conclusion that synaptic phenotypes in the *Fmr1*^*-/y*^ mice are not a consequence of altered glutamate signaling *per se*, but due to defective regulation of activity-dependent protein synthesis. Identification of proteins at synapses that can be substantially depleted by acute inhibition of protein synthesis and are necessary co-factors for functional and structural plasticity in WT mice may yield important insights into the pathophysiology of fragile X and related developmental disorders. Further, our data indicate that *metabotropic* NMDAR signaling might be an interesting target of opportunity for development of novel therapeutics to correct synaptic pathophysiology in fragile X [75].

## Supplementary information

Supplemental Figures and Legends
