# Supplementary material for: Dissociation of functional and structural plasticity of dendritic spines during NMDAR and mGluR-dependent long-term synaptic depression in wild-type and fragile X model mice
Source: Mol Psychiatry. 2020 Jul 1;26(9):4652–69. doi: 10.1038/s41380-020-0821-6 (PMC8095717; doi:10.1038/s41380-020-0821-6)
Supplement: Supplementary file 1 — Supplemental Figures and Legends [file 41380_2020_821_MOESM1_ESM.pdf]

Fig. S1- Thomazeau, Bosch et al.

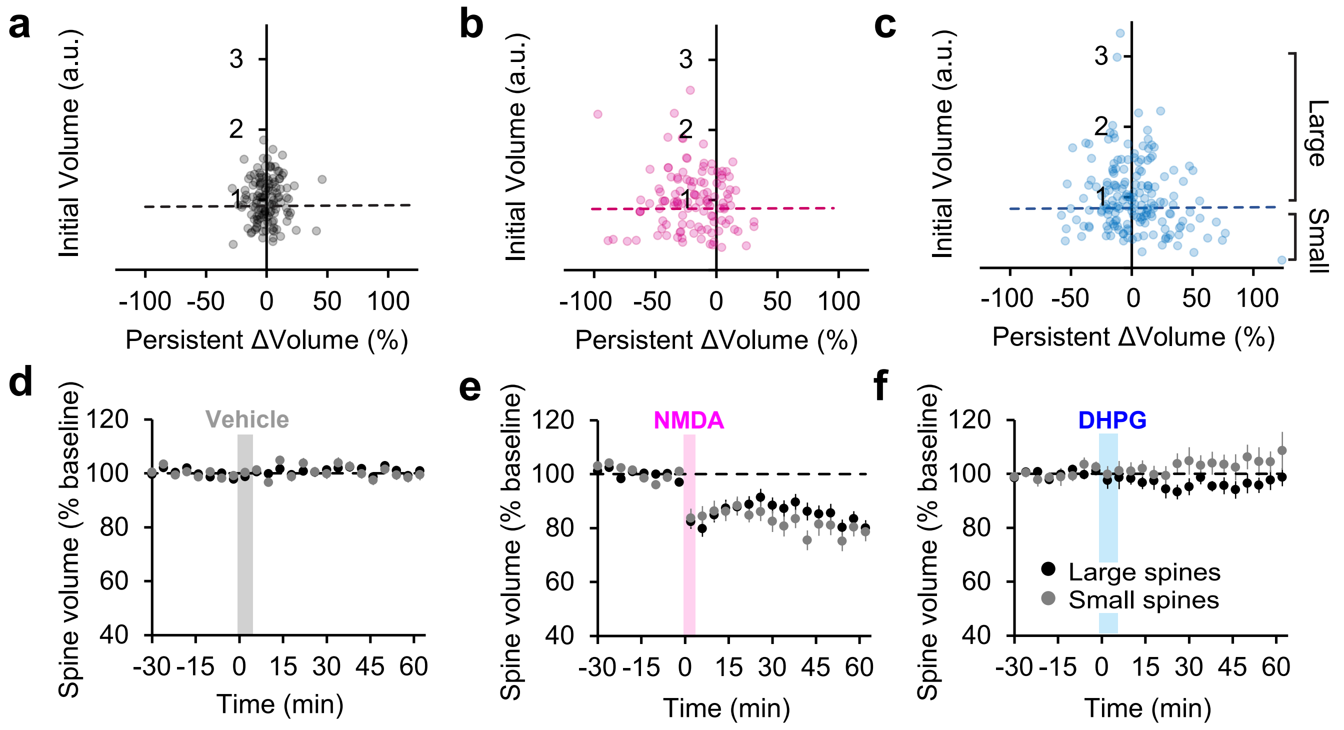

Figure S1. Morphological analysis of individual spines shows no correlation between initial spine size and persistent structural plasticity

a-c, The persistent change in spine volume (% of baseline) at the 50-60 min interval of each individual spine is plotted against their initial spine volume (in arbitrary units, a.u.) for control (**a,** n=130 spines; $R^2 = 0.0016$), NMDAR-LTD (**b,** n=118: $R^2 = 0.0021$) and mGluR-LTD (**c**, n=126; $R^2 = 0.0342$) experiments. No correlation was found. Spines are classified into two populations according to their initial size: larger ("Large") or smaller ("Small") than the median spine volume calculated for each experiment. **d-f**, Time course of spine volume (normalized to baseline) of these two populations of spines. Spines shrank after NMDA application (**e**) and did not shrink in control condition (**d**) or after DHPG application (**f**), regardless of their initial size prior to LTD induction.

Fig. S2- Thomazeau, Bosch et al.

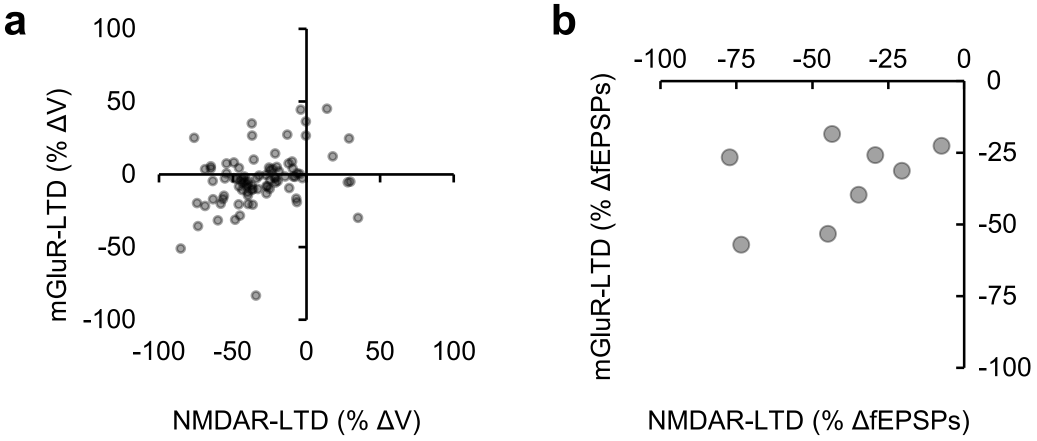

Figure S2. Individual spine shrinkage and fEPSP slope responses show no correlation between mGluR-LTD and NMDAR-LTD induced sequentially

a, Correlation plot between the percentage of spine volume change after DHPG application (mGluR-LTD) and the percentage of spine volume change after the subsequent application of NMDA on the same set of spines (NMDAR-LTD) for each individual spine (n=86; R^2 = 0.117) from experiments shown in **Fig. 1gB**. **b,** Correlation plot between the percentage of fEPSP slope responses change after DHPG application (mGluR-LTD) and the percentage of fEPSP slope responses change after the subsequent application of NMDA during the same recording (NMDAR-LTD) for each individual slice (n=8; R^2 = 0.1745) from experiments shown in **Fig. 1gA**.

Fig. S3- Thomazeau, Bosch et al.

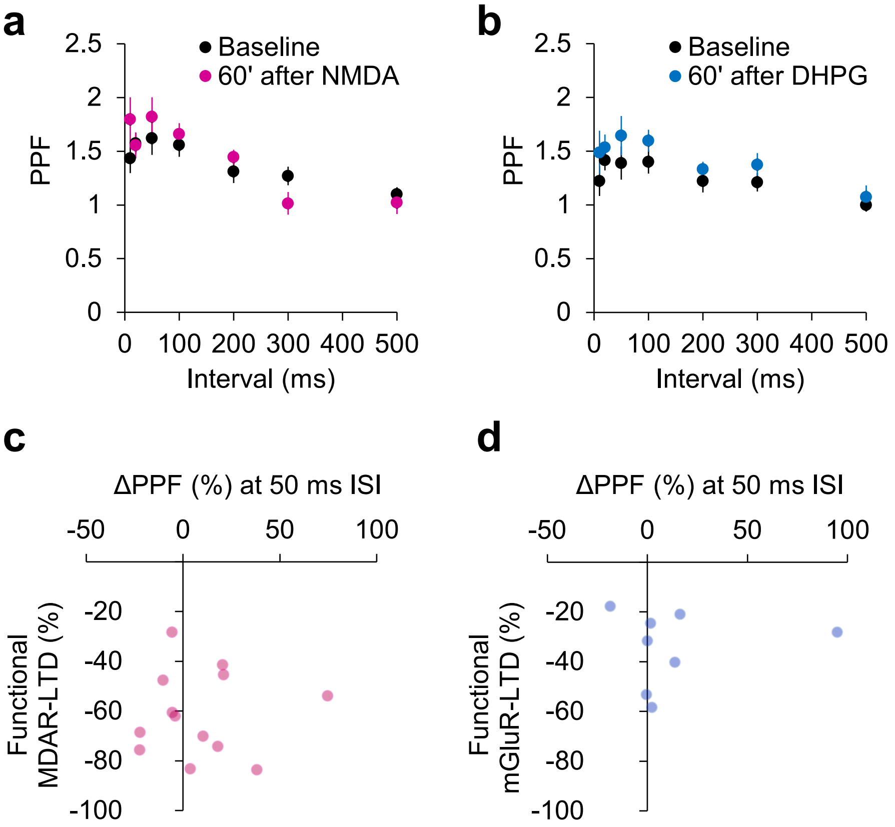

Figure S3. No presynaptic component in NMDAR-LTD and mGluR-LTD

a-b, The presynaptic contribution to LTD was assayed using pair-pulse facilitation (PPF) at different interstimulus intervals (ISI). No difference in the EPSP ratio of first versus second response was observed between baseline (black circles) and 60 min after application of NMDA (magenta circles, **a**) or DHPG (blue circles, **b**). **c-d,** No correlation was detected between the magnitude of structural LTD (percentage of spine volume change with respect to baseline) and the magnitude of paired-pulse facilitation (PPF) (percentage of change in EPSP ratio between baseline and 60 min post LTD induction at 50 ms interstimulus interval), either after NMDA (**c**, n=13) or DHPG (**d**, n=8) application.

Fig. S4- Thomazeau, Bosch et al.

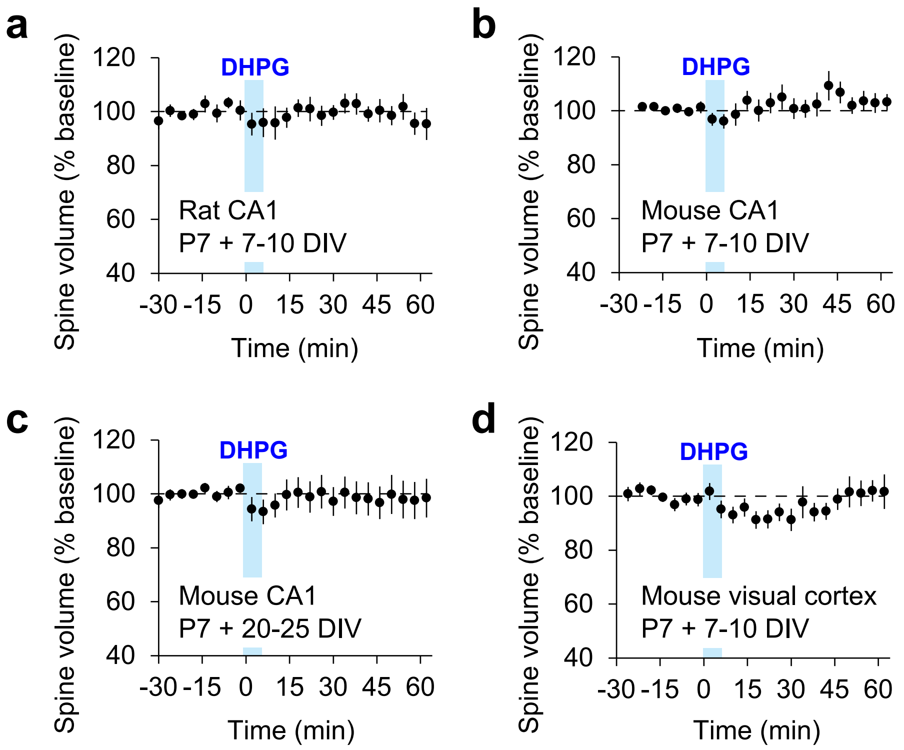

Figure S4. Absence of spine shrinkage during mGluR-LTD in different experimental systems

Time course of spine volume (normalized to baseline) after DHPG application in organotypic slice cultures obtained from P7 rat hippocampi (cultured for 7-10 DIV; **a**), obtained from P7 mouse hippocampi (cultured for 7-10 DIV; **b**), obtained from P7 mouse hippocampi (cultured for 20-25 DIV; **c**), or obtained from P7 mouse visual cortex (cultured for 7-10 DIV; **d**).

Fig. S5- Thomazeau, Bosch et al.

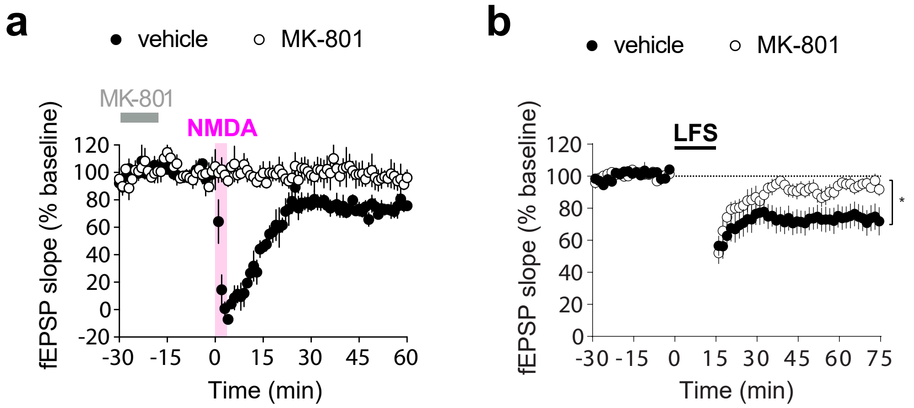

Figure S5. Application of a NMDAR open-channel blocker is sufficient to block LTD in WT mice

a, Time-course of averaged fEPSP slope responses in CA1 region of hippocampal slices from WT mice. A brief application of the NMDAR open-channel blocker MK-801 (40 µM), to prevent ion flux through receptors activated by electrical stimulation during baseline, inhibits functional LTD induced by NMDA (vehicle, black circles: 74.05 ± 3.33%, n=3; MK-801, white circles: 95.52 ± 5.34%, n=3; *p=0.0270, unpaired t-test). **b,** MK-801 (40 µM) also prevents induction of NMDA-LTD induced by a low frequency stimulation (LFS) protocol consisting in electrical stimulation for 15min @ 1Hz (vehicle, black circles: 73.40 ± 8.92%, n=6; MK-801, white circles: 95.86 ± 4.75%, n=6; *p=0.035 with respect to vehicle, unpaired t-test).

Fig. S6- Thomazeau, Bosch et al.

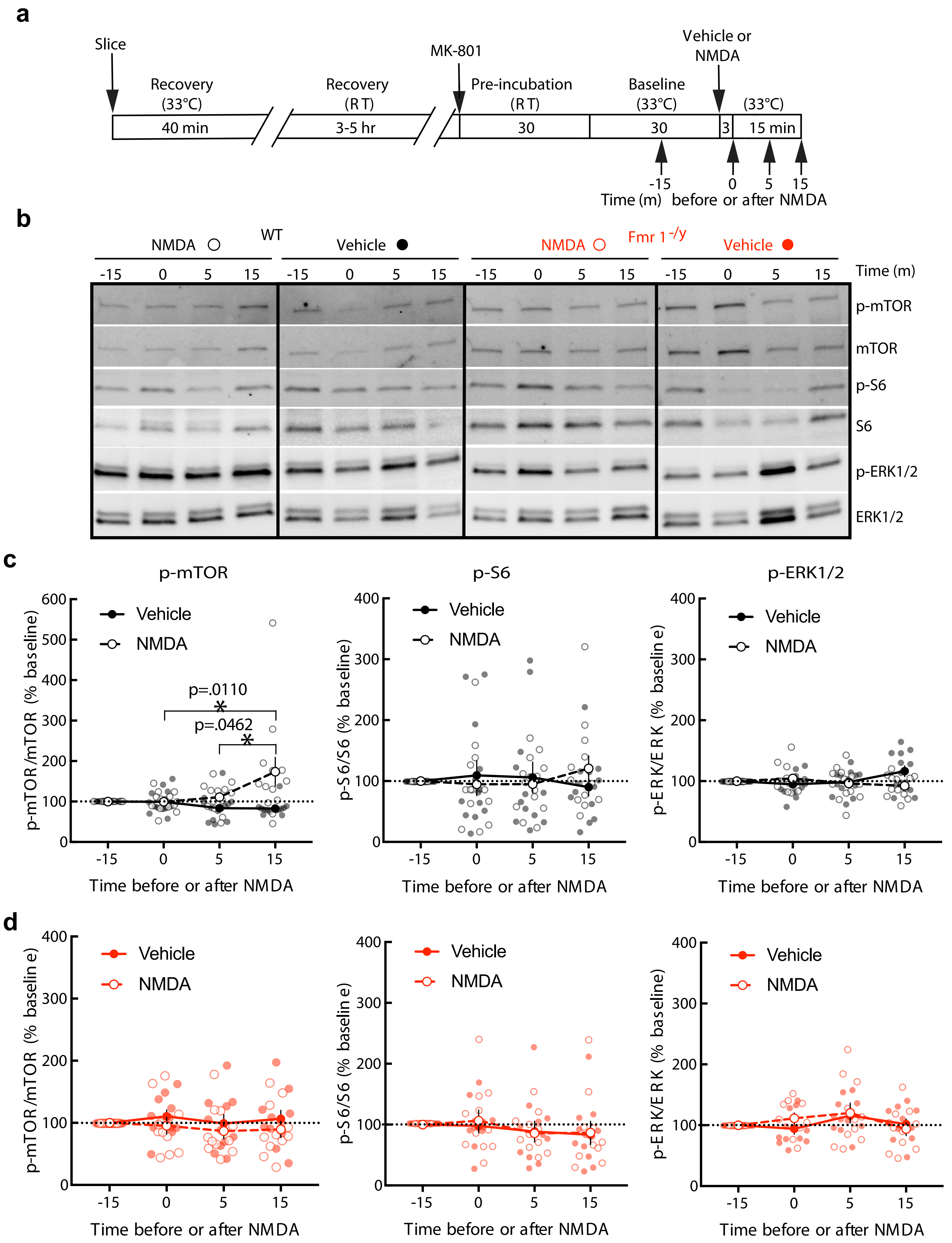

Figure S6. Brief application of NMDA in the presence of MK-801 fails to activate mTOR during spine structural plasticity

a, Experimental design showing time points and incubation times for biochemistry experiments in slices from WT (n=13) and *Fmr1*$^{-/y}$ (n=11) mice. **b,** Representative western blots of 4 time points for each antibody, treatment and genotype. **c-d**, Time course (normalized to baseline) of phosphorylated mTOR, S6 and ERK1/2 normalized to total amount of each protein from WT (**c**) and *Fmr1*$^{-/y}$ (**d**). An increase in mTOR phosphorylation is observed only 15 min after NMDA treatment and only in WT slices, when spine structural changes have already stabilized (two-way repeated measures ANOVA, time versus treatment, provided significant interaction: F=3.639, *p=0.0217. Post-hoc Bonferroni t-test revealed *p=0.011 at 15 min versus 0 min after NMDA treatment and *p=0.0462 15 min versus 5 min after NMDA treatment). WT vehicle, black circles; WT NMDA, open black circles; *Fmr1*$^{-/y}$ vehicles, red circles; and *Fmr1*$^{-/y}$ NMDA, open red circles.

Fig. S7- Thomazeau, Bosch et al.

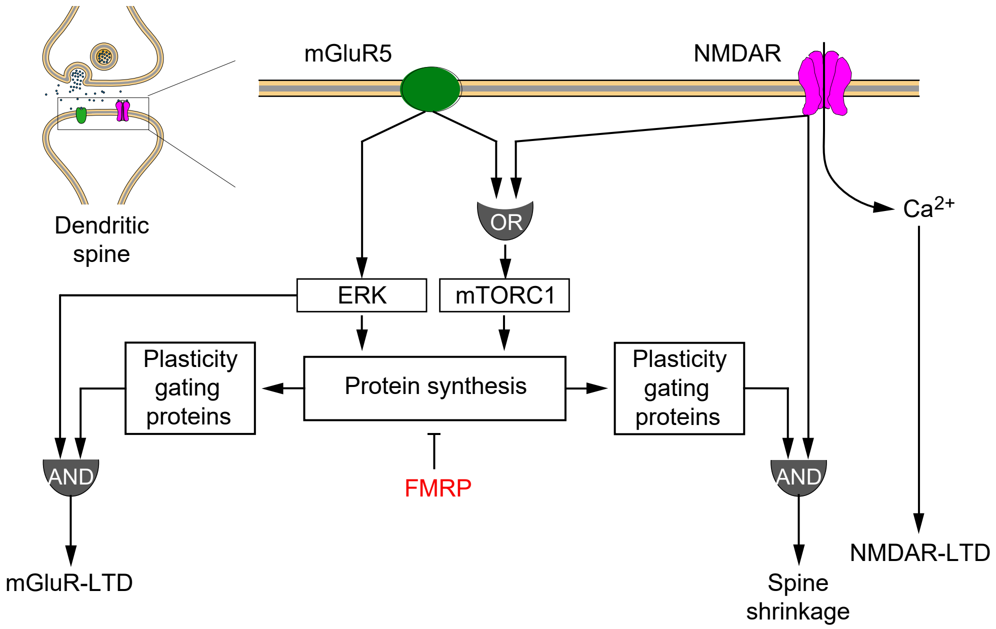

Figure S7. Unified model to account for the role of protein synthesis in functional and structural plasticity of dendritic spines in WT and *Fmr1$^{-/y}$* hippocampi. In WT mice, functional plasticity induced by activating mGluR5 (mGluR-LTD) and structural plasticity induced by metabotropic NMDAR activation (spine shrinkage) both require new protein synthesis downstream of ERK and mTOR signaling, respectively. In contrast, functional plasticity induced by Ca^{2+} flux through NMDARs (NMDAR-LTD) is mechanistically dissociable and does not require new protein synthesis. Unlike WT, in the *Fmr1$^{-/y}$* hippocampus neither mGluR-LTD nor NMDA-induced spine shrinkage require acute stimulation of protein synthesis by glutamate receptor activation. This finding suggests that "plasticity gating proteins", normally rate-limiting in WT, are constitutively increased in FX.
